# Antibiotics cause metabolic changes in mice primarily through microbiome modulation rather than behavioral changes

**DOI:** 10.1371/journal.pone.0265023

**Published:** 2022-03-17

**Authors:** Kale S. Bongers, Roderick A. McDonald, Katherine M. Winner, Nicole R. Falkowski, Christopher A. Brown, Jennifer M. Baker, Kevin J. Hinkle, Daniel J. Fergle, Robert P. Dickson

**Affiliations:** 1 Division of Pulmonary and Critical Care Medicine, Department of Internal Medicine, University of Michigan Health System, Ann Arbor, Michigan, United States of America; 2 Department of Microbiology and Immunology, University of Michigan Medical School, Ann Arbor, Michigan, United States of America; 3 Institute for Research on Innovation and Science, Institute for Social Research, University of Michigan, Ann Arbor, Michigan, United States of America; 4 Weil Institute for Critical Care Research and Innovation, Ann Arbor, Michigan, United States of America; University of Iowa, UNITED STATES

## Abstract

**Background:**

The microbiome is an important and increasingly-studied mediator of organismal metabolism, although how the microbiome affects metabolism remains incompletely understood. Many investigators use antibiotics to experimentally perturb the microbiome. However, antibiotics have poorly understood yet profound off-target effects on behavior and diet, including food and water aversion, that can confound experiments and limit their applicability. We thus sought to determine the relative influence of microbiome modulation and off-target antibiotic effects on the behavior and metabolic activity of mice.

**Results:**

Mice treated with oral antibiotics via drinking water exhibited significant weight loss in fat, liver, and muscle tissue. These mice also exhibited a reduction in water and food consumption, with marked variability across antibiotic regimens. While administration of bitter-tasting but antimicrobially-inert compounds caused a similar reduction in water consumption, this did not cause tissue weight loss or reduced food consumption. Mice administered intraperitoneal antibiotics (bypassing the gastrointestinal tract) exhibited reduced tissue weights and oral intake, comparable to the effects of oral antibiotics. Antibiotic-treated germ-free mice did not have reduced tissue weights, providing further evidence that direct microbiome modulation (rather than behavioral effects) mediates these metabolic changes.

**Conclusions:**

While oral antibiotics cause profound effects on food and water consumption, antibiotic effects on organismal metabolism are primarily mediated by microbiome modulation. We demonstrate that tissue-specific weight loss following antibiotic administration is due primarily to microbiome effects rather than food and water aversion, and identify antibiotic regimens that effectively modulate gut microbiota while minimizing off-target behavioral effects.

## Introduction

The microbiome is an important and increasingly-studied contributor to organismal metabolism. Studies have suggested that the composition of gut bacterial communities may affect obesity, skeletal muscle growth, and the development of diabetes [1–7]. Further study of the dynamic interplay between microbial and host metabolism holds promise to advance our understanding of human metabolic function and dysfunction.

Investigation of the microbiome’s role in host metabolism requires experimental manipulation of the body’s microbial communities, yet such methods of microbiome modulation remain limited. Germ-free and gnotobiotic animals offer investigators complete control over the body’s microbial communities, yet are expensive, difficult to maintain, and not widely available [8]. As such, many investigators use antibiotics as an inexpensive and easily-accessible method of microbiome modulation in animal models [8]. However, antibiotics commonly have off-target effects, including food and water aversion, that may indirectly influence metabolism [9,10]. These off-target effects may confound studies of the microbiome’s role in metabolism, obfuscating the true effects of microbiome modulation on organismal metabolism. The relative influence of these off-target effects of antibiotic administration on host metabolism, behavior, and the microbiome are unknown.

**Fig 1:**
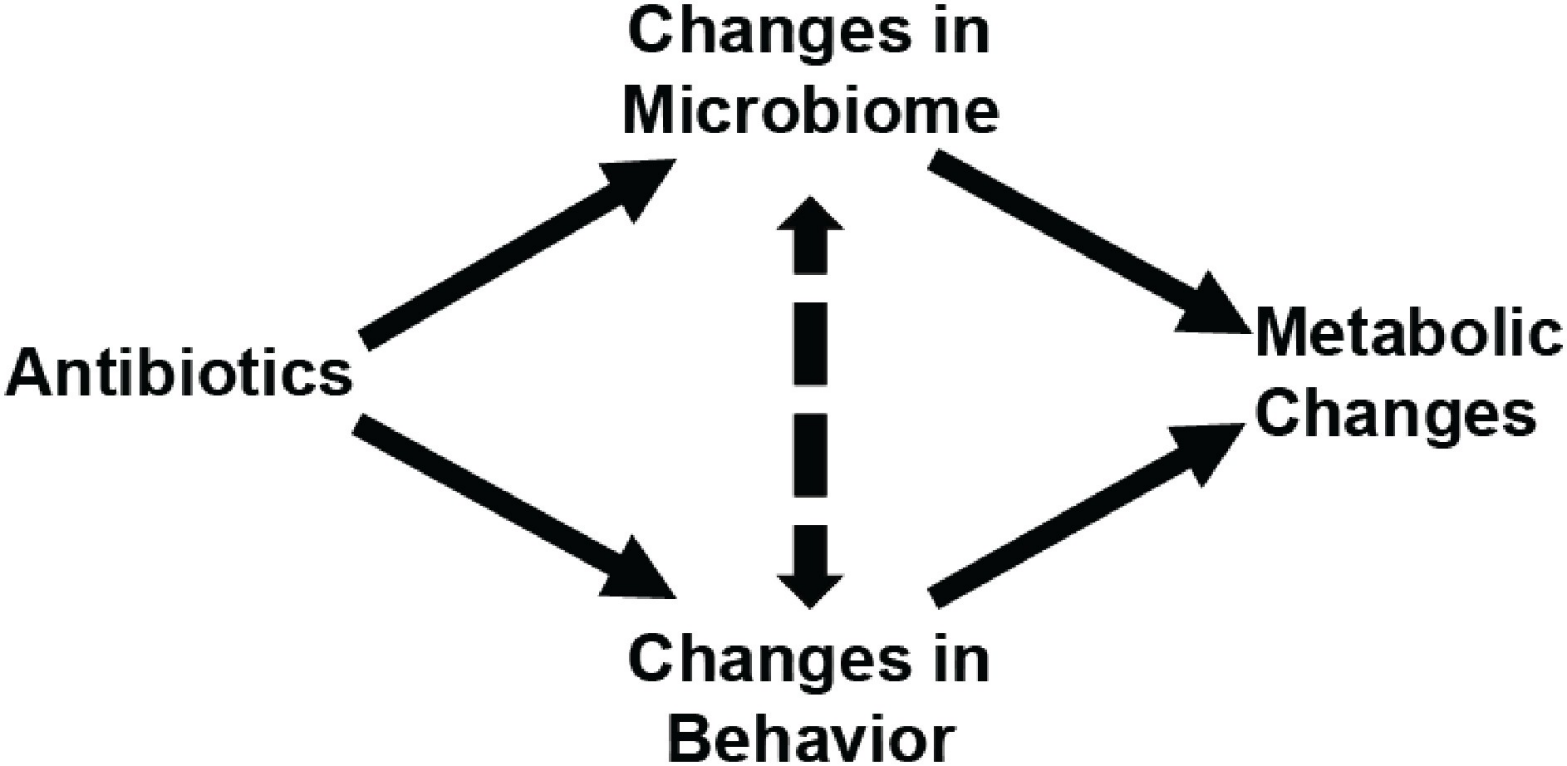
Potential pathways by which antibiotics may lead to metabolic changes.

We therefore designed a series of experiments to determine the relative influence of behavioral aversion and microbiome modulation in driving metabolic tissue weight changes in healthy, antibiotic-treated mice. A conceptual model of potential pathways by which antibiotics may alter host metabolism is presented in Fig 1.

## Materials and methods

### Ethics approval and consent to participate

No human data or specimens were used in this study. The University Committee on the Care and Use of Animals at the University of Michigan (PRO00007791) approved the animal studies. Laboratory animal care policies at the University of Michigan follow the Public Health Service policy on Humane Care and Use of Laboratory Animals.

### Mouse protocols

Conventional mice were 6-8-week old female specific pathogen-free C57BL/6 mice obtained from Jackson Laboratories (Bar Harbor, ME, USA). A list of organisms excluded from these mice can be found online at https://www.jax.org/jax-mice-and-services/customer-support/customer-service/animal-health/list-of-agents-monitored. Germ-free mice were 6–8 week old male and female C57BL/6 mice (both sexes were used due to limited mouse availability) obtained from the University of Michigan Germ-Free Mouse Core (Ann Arbor, MI, USA). Germ-free mice were housed in Tecniplast Iso-Positive Cages (Tecniplast, West Chester, PA, USA) under sterile conditions with sterile food, water, and bedding, and subject to regular culture and periodic control necropsies to ensure germ-free status in accordance with Germ-Free Mouse Core protocols. Unless otherwise noted, mice were housed in static microisolator colony cages at 21°C with a 12:12-hour light-dark cycle and had *ad libitum* access to water and standard chow (Envigo Teklad, Indianapolis, IN, USA). In metabolic caging experiments, mice were singly housed in metabolic cages (Tecniplast, West Chester, PA, USA), and food and water (either antibiotic-containing or control) were provided. Water and food were changed at minimum every 7 days. The Institutional Animal Care and Use Committee of the University of Michigan approved all protocols.

### Antibiotic experiments

For enteral antibiotic experiments, mice were given distilled water (control; Gibco Distilled Water, ThermoFisher Scientific, Waltham, MA, USA), distilled water with dissolved cefoperazone (0.5 g/L, Sigma-Aldrich, St. Louis, MO, USA), distilled water with dissolved enrofloxacin and ampicillin (0.27 g/L enrofloxacin, ThermoFisher Scientific, Waltham, MA, USA; 1 g/L ampicillin, Sigma-Aldrich, St. Louis, MO, USA), or distilled water with dissolved 1 g/L neomycin (Sigma-Aldrich, St. Louis, MO, USA), 1 g/L ampicillin (Sigma-Aldrich, St. Louis, MO, USA), 1 g/L metronidazole (Sigma-Aldrich, St. Louis, MO, USA), and 0.5 g/L vancomycin (Sigma-Aldrich, St. Louis, MO, USA) for eight days. For intraperitoneal injection experiments, mice were given intraperitoneal injections of 50 mg/kg ceftriaxone in 200 μl sterile saline, 200 μl sterile saline alone (sham), or no injection (control) for four days. At the conclusion of the experiments, mice were necropsied.

### Bitterant experiments

Mice were given distilled water (control; Gibco Distilled Water, ThermoFisher Scientific, Waltham, MA, USA), distilled water with dissolved 3 mM denatonium benzoate (Sigma-Aldrich, St. Louis, MO, USA), or distilled water with 75 mM salicin (Sigma-Aldrich, St. Louis, MO, USA).

### Mouse NMR analysis

Mouse body composition was determined serially with an EchoMRI 1100 (EchoMRI, Houston, TX, USA) as described previously [11].

### Tissue collection and processing

Mice were sacrificed via CO_2_ asphyxiation, and tissues were harvested in this order: cecum, liver, perigonadal fat pads, kidneys, retroperitoneal fat pads, tibialis anterior muscle, gastrocnemius-soleus complex muscles. Cecum was harvested with sterile technique, with instruments rinsed in ethanol and flamed before each harvest. Cecal tip samples were snap-frozen using liquid nitrogen and stored at -80°C until DNA isolation. All samples not designated for microbiome analysis were collected by a separate dissector using non-sterile technique, weighed, and then snap-frozen.

### Bacterial DNA isolation

Genomic DNA was extracted from mouse tissue using DNeasy Blood and Tissue kit (Qiagen, Hilden, Germany) and homogenized in PowerBead tubes (Qiagen, Hilden, Germany) as previously described [12,13]. One kit was used for all cefoperazone and enrofloxacin/ampicillin studies and controls; a second kit was used for all four-drug regimen studies and controls and all itterant studies and controls.

### Bacterial DNA quantification

Bacterial DNA was quantified with a QX200 Droplet Digital PCR (BioRad, Hercules, CA, USA, catalog no. 1864003). Primers and cycling conditions were as described previously [12,13]. Droplets were quantified using BioRad QuantaSoftTM Analysis Pro software, version 1.0.596.

### 16S rRNA gene sequencing

The V4 region of the 16S rRNA gene was amplified using published primers, as described previously [12]. Sequencing was performed using the Illumina MiSeq platform (San Diego, CA, USA), according to the manufacturer’s instructions with modifications as described previously [12]. AccuPrime High-Fidelity Taq was used; the PCR cycling conditions were 95°C for 2 minutes, followed by 20 cycles of touchdown PCR, then 20 cycles of standard PCR, and finishing at 72°C for 10 minutes [12]. Empty wells, sterile water, and DNA isolation controls were used as negative controls, while synthetic standard communities (Zymo Research, Irvine, CA, USA, catalog no. D6306) were used as positive controls. FASTQ files were generated with paired end reads and retained for further analysis.

### Adequacy of sequencing

We generated 1,488,408 bacterial reads among the cecal samples, with a mean ± SD of 1,5034 ± 12,058 reads per specimen with a range of 187–60,175 reads per specimen. None of the cecal samples were excluded from analysis due to insufficient depth of sequencing.

### Data analysis

Sequence data were processed and analyzed using *mothur* software according to the standard operating protocol for MiSeq sequence data (https://mothur.org/wiki/miseq_sop/); minimum sequence length was 250 base pairs [12,13]. A shared community file and phylotypes file were generated, using operational taxonomic units (OTUs) binned at 97% identity in mothur (version 1.43.0) [12]. We performed ordinations using principal component analysis in R (version 4.0.2) on Hellinger-transformed normalized OTU tables as described previously [12], and compared community composition differences using PERMANOVA (via the *adonis* function in the *vegan* package) with 10,000 permutations using Euclidean distances. For non-microbiome mouse work we used paired two-tailed t-tests to compare repeated measures in the same animals, and unpaired two-tailed t-tests for all other two-group comparisons. We used one-way ANOVA with Dunnett’s post hoc test for 3 or more groups where we compared each experimental treatment to control, and Tukey’s post hoc test for multiple comparisons among all groups. All tests used P = 0.05 as a threshold for significance.

## Results

### Commonly-used enteral antibiotic regimens have profound but highly variable effects on host metabolism and gut microbiota

**Fig 2:**
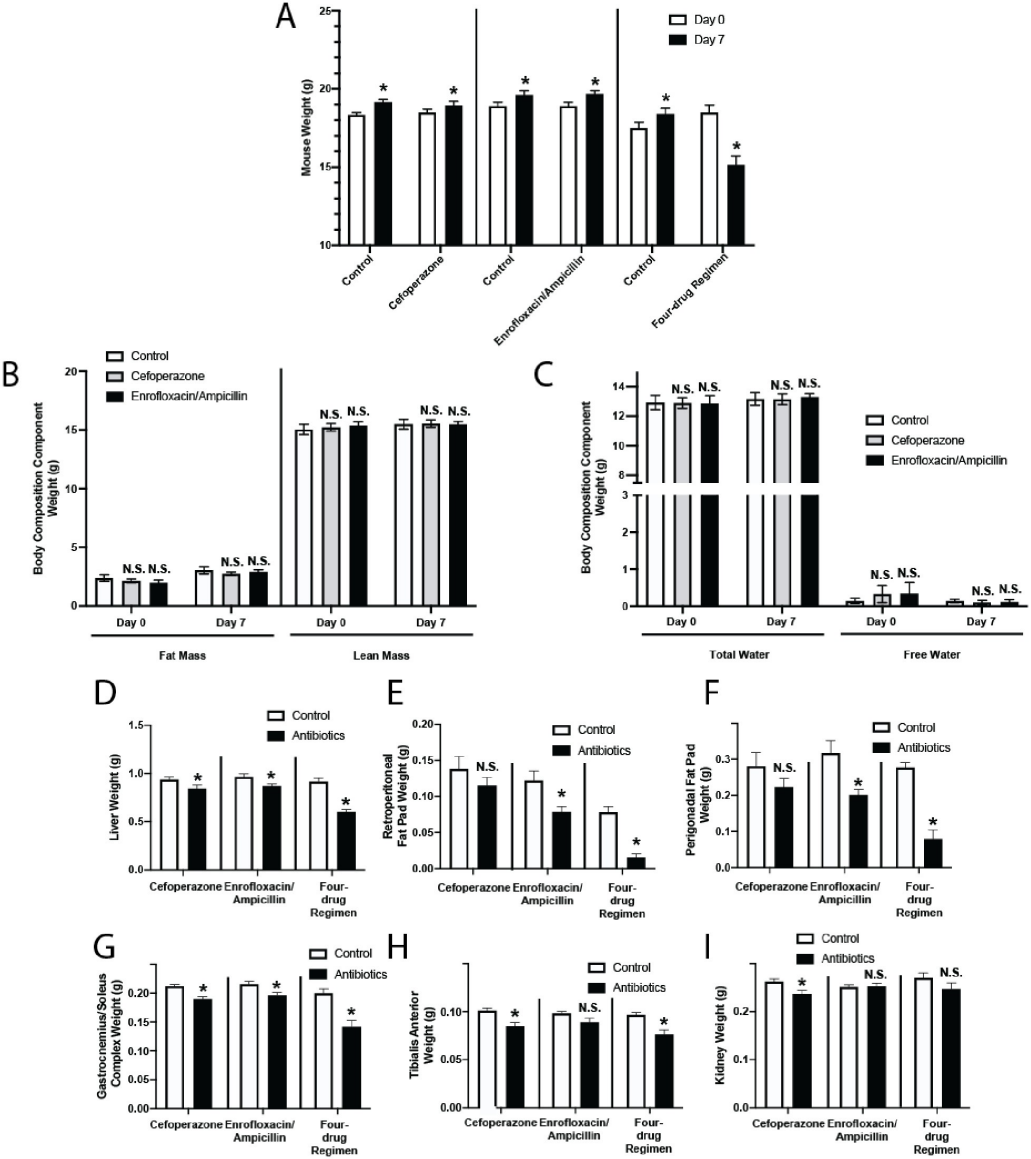
Commonly administered enteral antibiotic regimens have profound yet variable effects on host metabolism. Mice were given an oral regimen of antibiotics: (1) 0.5 g/L cefoperazone; (2) 0.27 g/L enrofloxacin and 1 g/L ampicillin; (3) 1 g/L neomycin, 1 g/L ampicillin, 1 g/L metronidazole, and 0.5 g/L vancomycin (the “four-drug regimen”); in distilled drinking water. Control mice were given distilled drinking water alone. Mice were given *ad libitum* access to food and water. (A) Comparison of mouse body weights at 0 and 7 days. Error bars denote SEM. n = 10–20, * *P*<0.05. (B-C) NMR whole body composition analysis of mice treated with enrofloxacin/ampicillin and cefoperazone regimens. Mice were analyzed at day 0 (immediately prior to antibiotic administration) and day 7 (at the conclusion of antibiotic therapy). n = 10, N.S. = not significant, *P* > 0.05. (B) Fat mass and lean mass. (C) Total body water and free body water. (D-I) Mice were necropsied on day 8 of treatment and weights of liver (D), retroperitoneal fat pads (E), perigonadal fat pads (F), bilateral gastrocnemius-soleus complex muscles (G), bilateral tibialis anterior muscles (H), and bilateral kidneys (I) were compared to their respective controls. n = 10, * *P* < 0.05. Error bars denote SEM.

To determine the effects of antibiotics on overall mouse metabolism, we compared three different antibiotic regimens delivered enterally (via drinking water) to healthy mice over seven days: 1) cefoperazone, 2) enrofloxacin/ampicillin, and 3) a four-drug regimen of neomycin, vancomycin, metronidazole and ampicillin. All three regimens have been used in prior microbiome-related research [8,10,14,15]. We found that while mice treated with cefoperazone and enrofloxacin/ampicillin gained weight during the seven-day treatment period, mice receiving the four-drug regimen lost significant weight over the same duration, consistent with previous reports (Fig 2A) [9,10]. Next, we found that the two regimens that did not cause weight loss had no effects on mouse body composition with whole-animal NMR spectroscopy (Fig 2B and 2C), including lean mass, fat mass, and total and free body water.

However, as these weight and body composition findings could mask more subtle shifts in organ and tissue weights, we then evaluated whether antibiotic-treated mice had significant changes in specific organ and tissue weight compared to control mice. We compared post-treatment weights of internal organs (liver and kidneys), representative skeletal muscle groups (tibialis anterior [TA] and gastrocnemius-soleus complex [GSC] muscles), and representative fat pads (perigonadal and retroperitoneal fat pads). Skeletal muscle, liver, and fat are significant sources of metabolic stores [16]. We found that all three antibiotic regimens reduced the weights of the liver and GSC muscle, while two of the three antibiotic regimens reduced weights of the TA muscle and both fat pads, and one of the three regimens significantly reduced kidney weight (Fig 2D–2I). Most of these changes persisted even after normalizing tissue weights to body weights (S1 Fig). Taken together, these data demonstrated that even in models in which antibiotic-treated mice gain weight, antibiotic administration results in significant decreases in organ and tissue weights compared to control mice, confirming the tissue-specific effects of antibiotic administration.

**Fig 3:**
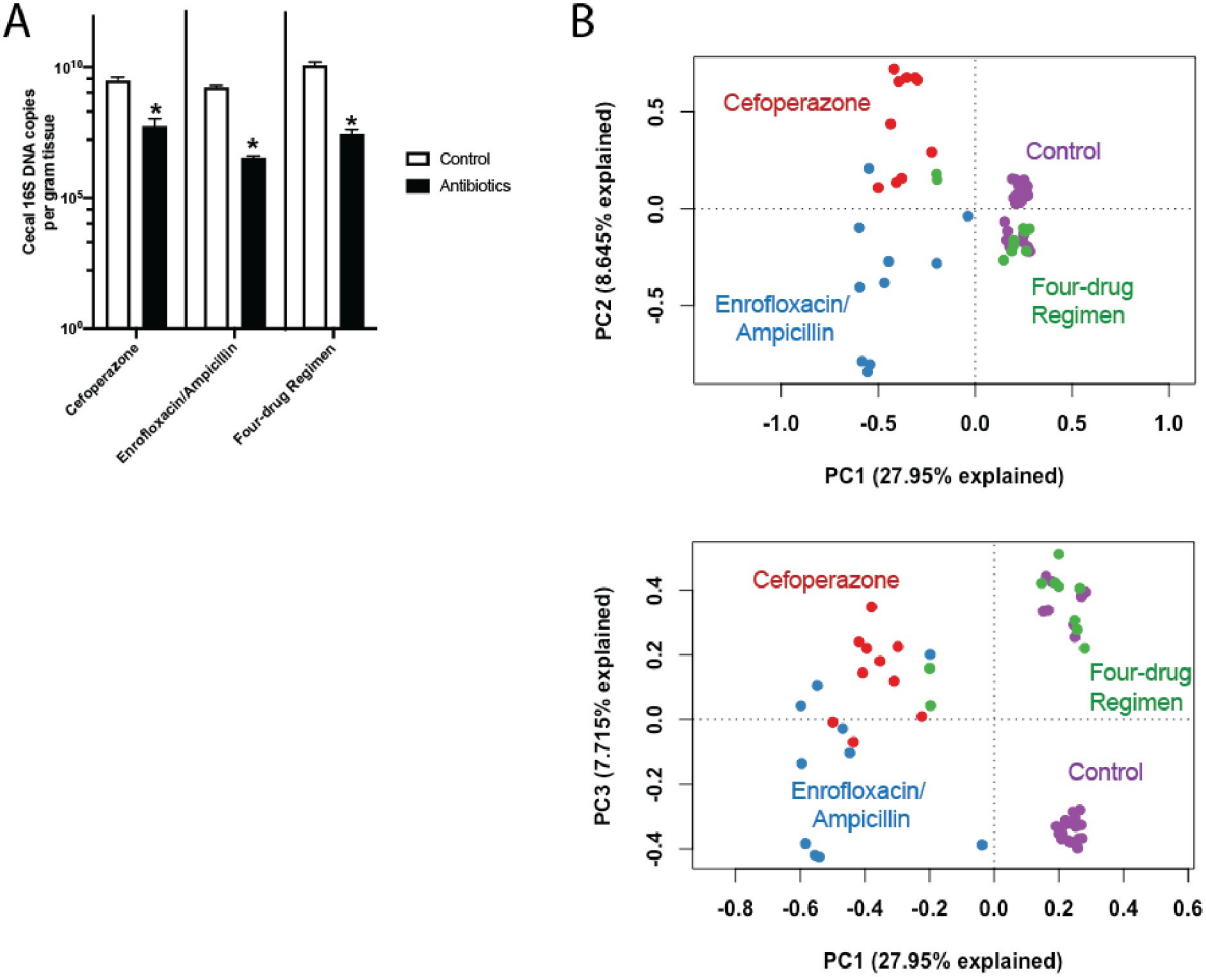
Effects of oral antibiotic regimens on cecal bacterial density and community composition. Mice were administered enteral antibiotics via distilled drinking water (cefoperazone, enrofloxacin/ampicillin, or the four-drug regimen); control mice were administered distilled drinking water alone. Mice were provided *ad libitum* access to food and water and harvested on day 8. (A) Comparison of 16S bacterial DNA copy number in mouse cecal tip. n = 10, * *P*<0.05. Error bars denote SEM. (B) PCA plots of microbiota in mouse cecum. Each dot represents one mouse. Gut communities of all antibiotic-treated groups were significantly distinct from those of control mice as well as each other (*P* < 0.0001 for all comparisons, PERMANOVA). *Top*: Graph of PC1 versus PC2. *Bottom*: Graph of PC1 versus PC3.

Observing these tissue weight changes, we then confirmed that these antibiotic regimens also caused concurrent, significant effects on the gut microbiome. As expected, each of the antibiotic regimens significantly reduced bacterial burden in the cecum (Fig 3A). Further, as characterized via 16S rRNA gene amplicon sequencing, each antibiotic regimen significantly altered the community composition of gut microbiota as compared to control mice (P<0.0001, PERMANOVA; Fig 3B). In addition, the gut communities of experimental groups differed from each other (P<0.0001, PERMANOVA; Fig 3B). Bacterial family composition in the cecum also varied with antibiotic treatment (S2 Fig). Taken together, this demonstrates that oral antibiotic treatment both changes density and composition of the mouse cecal microbiome, and that different antibiotic regimens cause distinct community changes within the gut microbiome.

### Oral antibiotic regimens cause food and water aversion in mice

**Fig 4:**
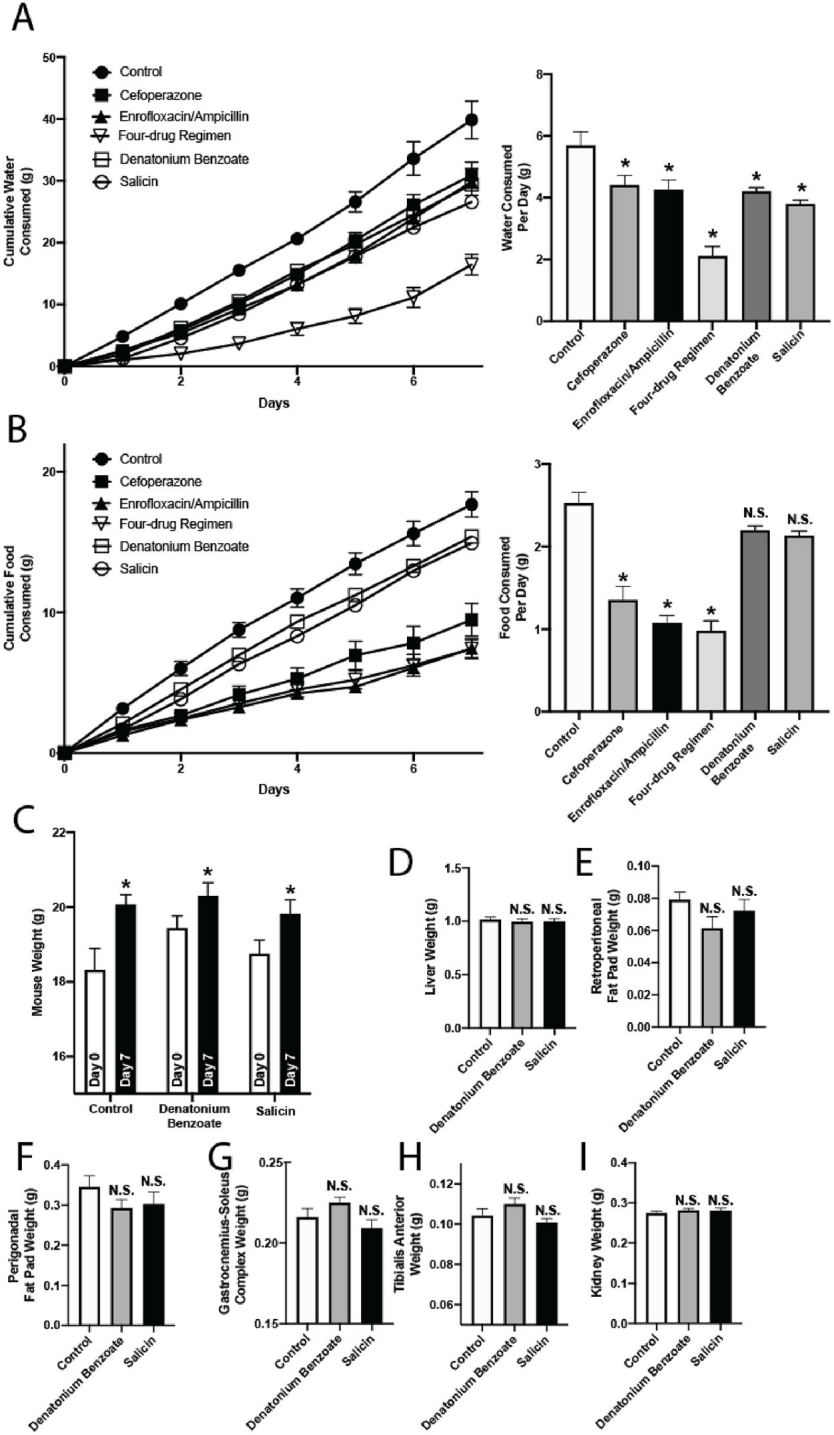
Antibiotic-treated mice exhibit reduced food and water consumption, and this is not explained by bitter taste alone. Mice were treated with cefoperazone, enrofloxacin/ampicillin, or the four-drug regimen in distilled water. Distilled water alone was used for control mice, and antimicrobially-inert but bitter-tasting compounds (3 mM denatonium benzoate or 75 mM salicin) in distilled water were used as positive controls. Mice were provided ad libitum access to food and water. (A) Water consumption. Error bars denote SEM. Left: Cumulative water consumption per mouse. Right: Average water consumed per mouse per day. n = 6–7, * *P*<0.05. (B) Food consumption. Error bars denote SEM. Left: Cumulative food consumption per mouse. Right: Average food consumed per mouse per day. n = 6–7, * *P*<0.05. (C-I) Mice were treated with denatonium benzoate or salicin in distilled water. (C) Mouse weights were compared at day 0 and day 7 for each treatment. n = 10, * *P* < 0.05. (D-I) Mice were sacrificed and subject to necropsy on day 8 of treatment and weights of liver (D), retroperitoneal fat pads (E), perigonadal fat pads (F), bilateral gastrocnemius-soleus complex muscles (G), bilateral tibialis anterior muscles (H) and kidneys (I) were compared. n = 10, N.S. denotes non-significance. Error bars denote SEM.

Having confirmed that enteral antibiotic regimens have significant effects on organ and tissue weights, we next tested whether these metabolic effects were attributable to food and water aversion. Some antibiotics, including metronidazole, which is included in the four-drug regimen at 8.5 mM concentration, taste bitter (for metronidazole, at concentrations around 1–10 mM) leading to reduced mouse oral intake [9,17]. To evaluate this possibility, we studied individually-housed mice in metabolic cages, which allow for evaluation of food and water consumption in a closed system. As an additional control, we gave separate cohorts of mice high doses of two separate bitter-tasting but antimicrobially-inert compounds in their drinking water. These bitterants, denatonium benzoate and salicin, have been well-studied and shown to be strongly aversive in mice at dosages below what we administered here [18–21]. Specifically, while denatonium has been shown to be aversive in the 0.01–1 mM range [18–20], we used a 3 mM concentration; while salicin has been shown to be aversive in the 10–50 mM range [18,20], we used a 75 mM concentration. As expected, all of the tested antibiotic or bitterant treatments significantly reduced water consumption (Fig 4A). Of note, mice administered the four-drug regimen consumed less water than even the bitterant-treated groups (P < 0.0001). Additionally, the antibiotic-treated mice also ate significantly less food (which contained no antibiotics or bitterants) than control-treated animals (Fig 4B). Unlike the antibiotic-treated mice, the bitterant-treated mice ate the same amount as control mice (Fig 4B). Despite drinking less, bitterant-treated mice gained significant weight over the course of their treatment (Fig 4C). Moreover, they did not exhibit any differences in tissue weights compared to control mice (Fig 4D–4I). Taken together, these findings suggest that the metabolic effects of enteral antibiotics are not entirely attributable to taste.

**Fig 5:**
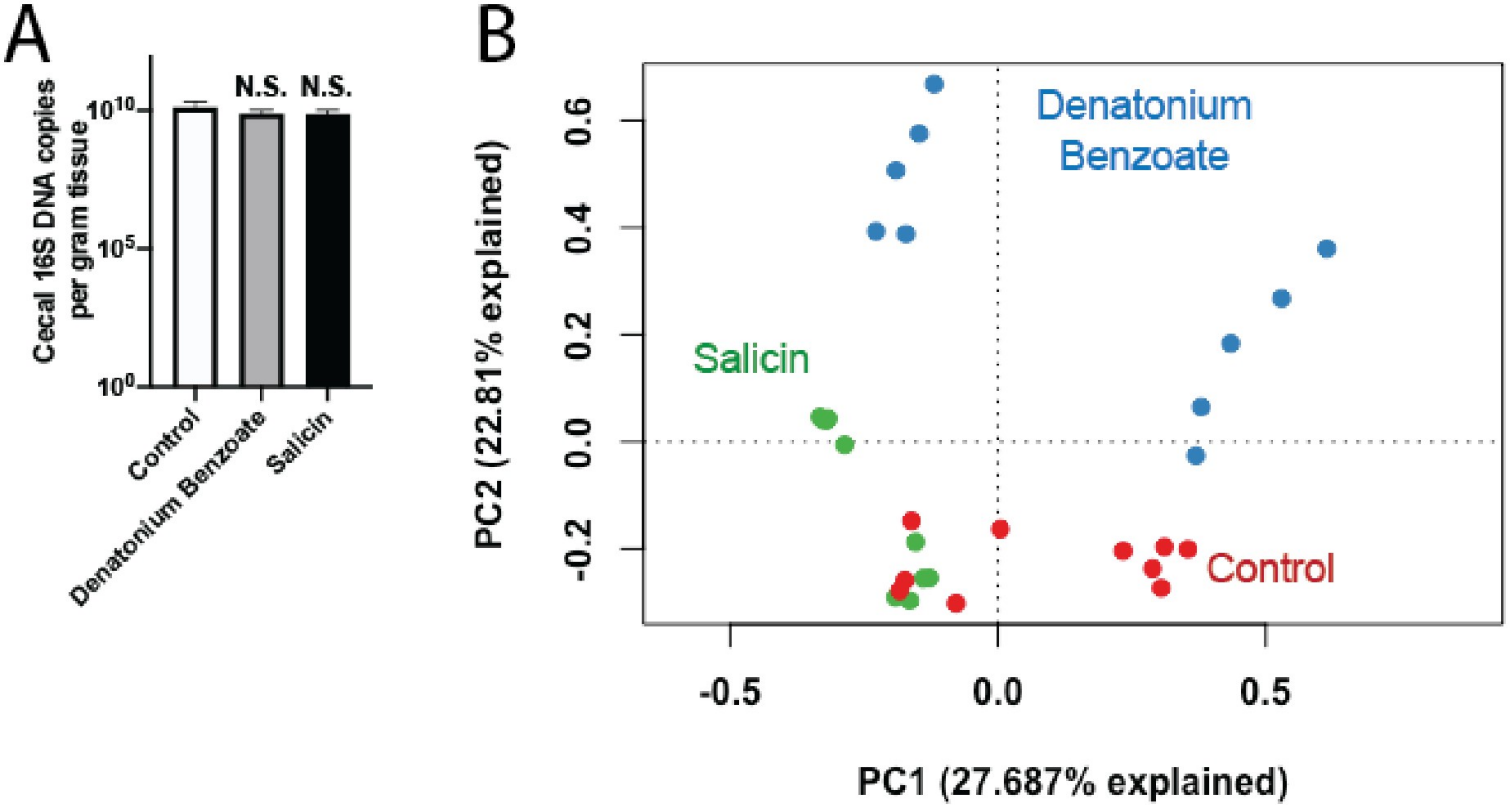
Effects of bitterants on cecal bacteria density and community composition. Mice were treated with antimicrobially-inert but bitter-tasting compounds (3 mM denatonium benzoate or 75 mM salicin) in distilled water, or distilled water alone, for 8 days. Mice were provided ad libitum access to food and water. (A) Comparison of 16S bacterial DNA copy number in mouse cecal tip. n = 10, * *P*<0.05. Error bars denote SEM. (B) PCA plot of microbiota in mouse cecum. Each dot represents one mouse. *P* < 0.0002 for all comparisons (PERMANOVA).

To confirm that bitterant treatments did not cause antimicrobial effects, we analyzed mouse cecal bacterial burden and found no significant differences between mice receiving either bitterant or control (Fig 5A). In contrast, bitterant-treated mice did exhibit significantly different cecal bacterial community composition as compared to controls (P < 0.0002; Fig 5B). This change in community composition is unsurprising, as previous studies have shown that changes in food and water consumption alone can change microbiome composition due to altered nutrient availability [22–24]. In addition, though they do not have known direct antimicrobial effects, there is some evidence that bitterants can modulate the host innate immune system [25,26]. Interestingly, the two tested bitterants also had distinct bacterial community compositions from each other (P < 0.0001; Fig 5B), suggesting that specific bitterant compounds may have different effects on the microbiome. In summary, these data demonstrate that, while water aversion may lead to effects on microbiome composition, the bitterants did not have the suppressive antimicrobial effects observed with the three tested antibiotic regimens.

### Systemically-administered antibiotic treatment also reduces mouse tissue weights and food and water consumption

**Fig 6:**
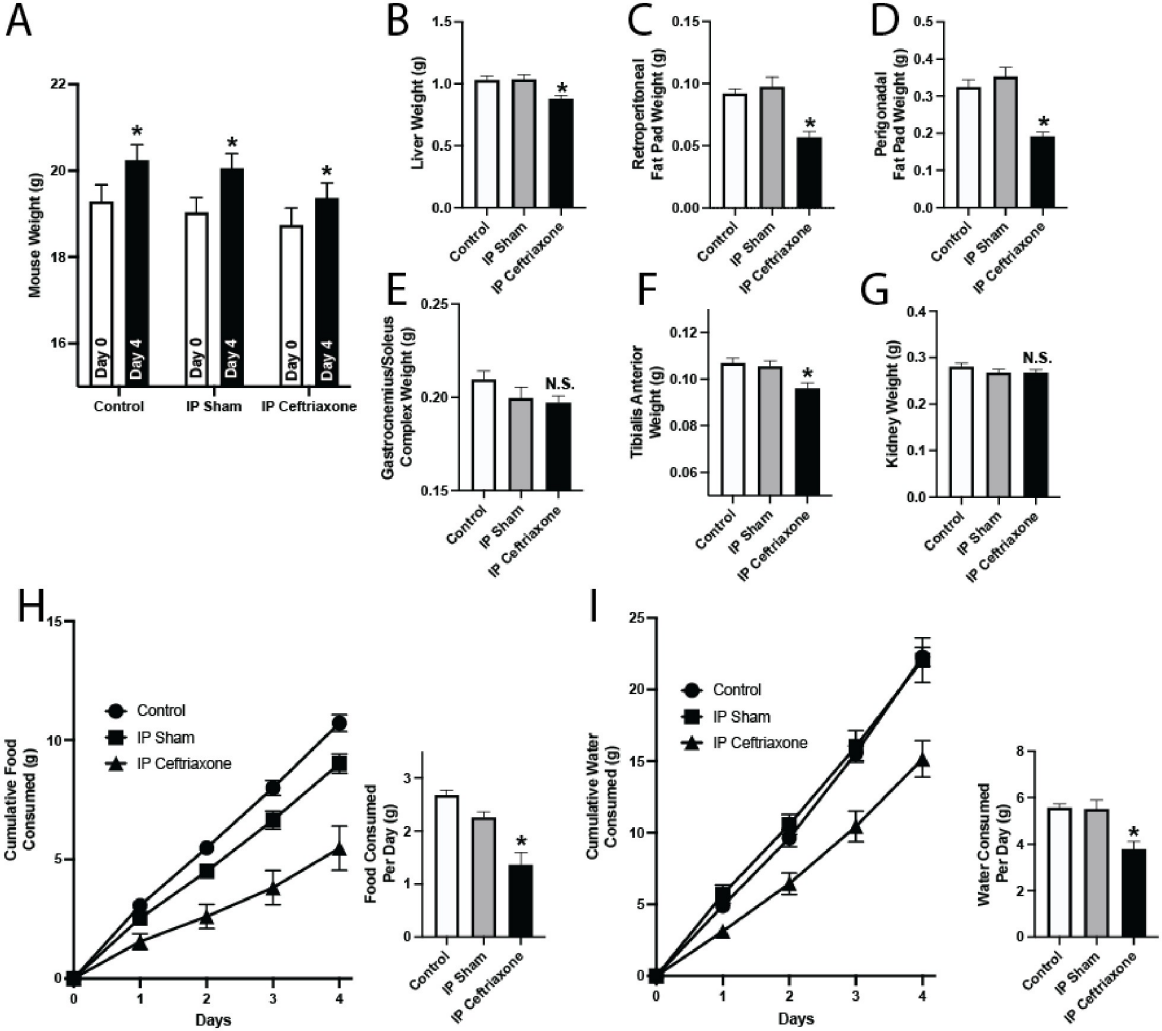
Intraperitoneal antibiotic administration reduces organ/tissue weight and food and water consumption. C57BL/6 mice were used as controls or given intraperitoneal injections of 200 μl saline (IP Sham) or 50 mg/kg ceftriaxone in 200 μl saline (IP Ceftriaxone) once daily for four days. (A-G) Mice were housed in normal cages. (A) Comparison of mouse body weights at 0 and 4 days. Error bars denote SEM. n = 10, * *P*<0.05. (B-G) Mouse organ and tissue weights at necropsy. Mice were necropsied at four days of treatment, and weights of liver (B), retroperitoneal fat pads (C), perigonadal fat pads (D), bilateral gastrocnemius-soleus complex muscles (E), bilateral tibialis anterior muscles (F), and kidneys (G) were compared. n = 10, * *P*<0.05. Error bars denote SEM. (H-I) Mice were placed in metabolic cages. (H) Food consumption. Error bars denote SEM. n = 6, * *P*<0.05. Left: Cumulative food consumption per mouse. Right: Average food consumed per mouse per day. (I) Water consumption. Error bars denote SEM. n = 6, * *P*<0.05. Left: Cumulative water consumption per mouse. Right: Average water consumed per mouse per day.

To more directly determine the effect of microbiome modulation on systemic metabolism independent of food and water aversion due to taste, we exposed healthy mice to a systemically-administered antibiotic regimen which bypasses the gastrointestinal tract and therefore the effect of taste on food and water consumption. We gave healthy mice four days of intraperitoneal injections with 50 mg/kg ceftriaxone, choosing this dose as it has been described previously in the literature to cause significant change in gut microbiome composition [12]. Ceftriaxone-treated mice gained weight (Fig 6A) and, as seen with oral antibiotic-treated mice, had significantly reduced liver, muscle, and fat weights (Fig 6B–6G). Interestingly, mice treated with intraperitoneal ceftriaxone had reduced food (Fig 6H) and water (Fig 6I) intake compared to control and sham-treated mice. These findings recapitulate our findings in oral antibiotic-treated mice (Figs 2, 4A and 4B), and provide additional evidence that antibiotics cause both metabolic changes and behavioral changes (such as food and water aversion) independent of the direct aversive effects of taste.

### Germ-free mice do not exhibit organ or tissue weight changes after oral antibiotic treatment

**Fig 7:**
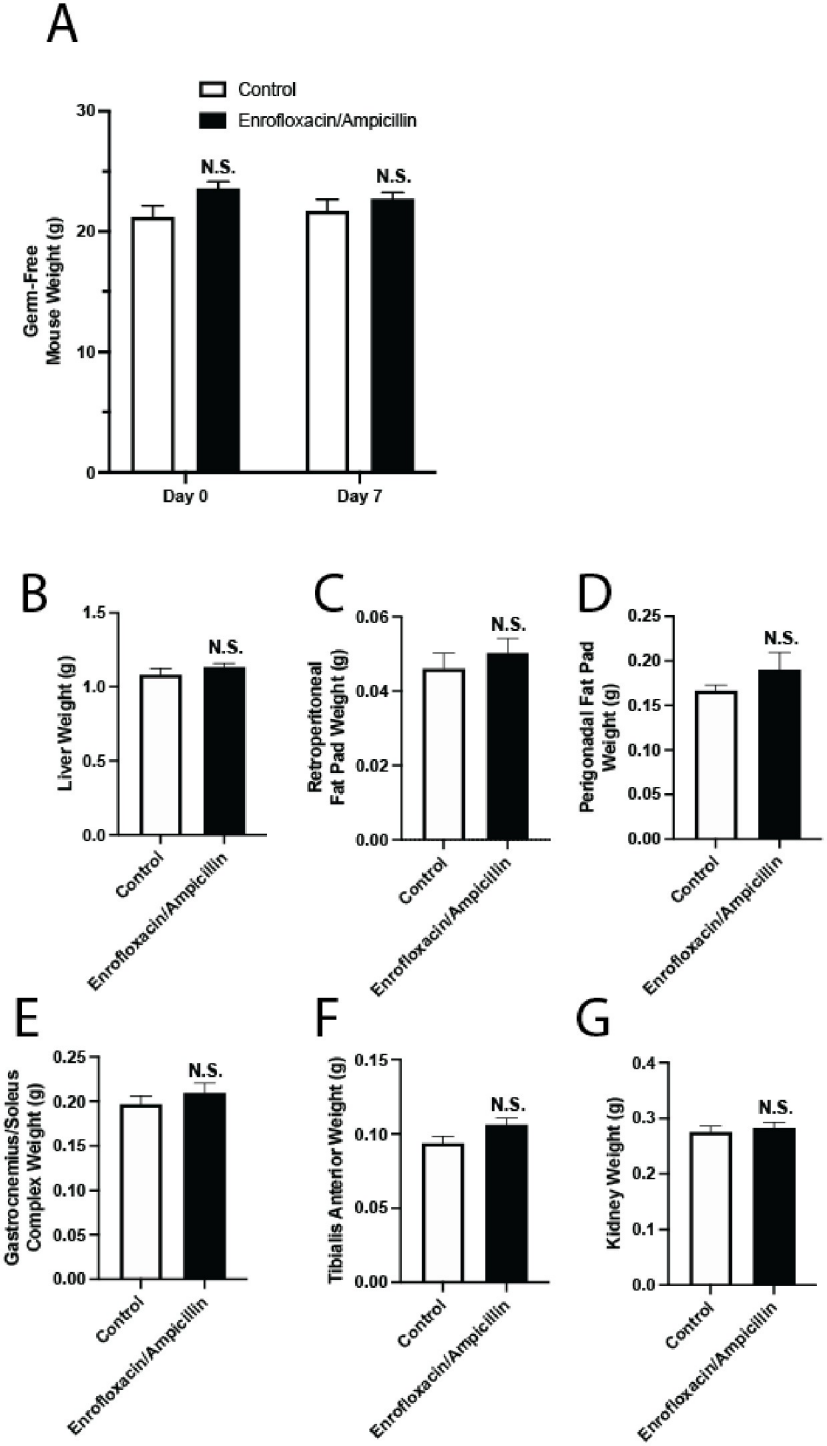
Oral antibiotic administration does not reduce organ/tissue weight in germ-free mice. Germ-free C57BL/6 mice were provided distilled drinking water, with or without 0.27 g/L enrofloxacin and 1 g/L ampicillin, for 8 days. (A) Comparison of mouse body weights at 0 and 7 days. Error bars denote SEM. n = 7, N.S. denotes non-significance, *P* > 0.05. (B-G) Mice were necropsied on day 8, and liver (B), retroperitoneal fat pads (C), perigonadal fat pads (D), bilateral gastrocnemius-soleus complex muscles (E), bilateral tibialis anterior muscles (F), and kidneys (G) were compared. Error bars denote SEM. n = 7, N.S. denotes non-significance, *P* > 0.05.

To confirm that the microbiome was indeed mediating these metabolic changes rather than an off-target effect of antibiotics, we next administered oral antibiotics to germ-free mice, which do not have a detectable microbiome. Unlike conventional mice treated with enrofloxacin/ampicillin (Fig 2), identically-treated germ-free mice did not lose liver, muscle, or fat weight compared to controls (Fig 7A–7G). We chose enrofloxacin/ampicillin in this model to avoid the known weight loss and food aversion evident in the four-drug regimen (Fig 2 and [9]). This provides additional evidence that antibiotic effects on the microbiome, not off-target behavioral effects on food and water consumption, cause these consistent metabolic changes.

## Discussion

In this study, we demonstrate the direct (microbiome-mediated) and indirect (behavioral) effects of antibiotics on mouse metabolism. We find that antibiotic-treated mice lose weight in their muscles, fat pads, and livers. Furthermore, while oral antibiotic-treated mice eat and drink less than control mice, we demonstrate using several lines of evidence that these metabolic changes are not due to food and water aversion alone, but primarily depend on microbiome modulation. First, using bitter-tasting but antimicrobially-inert compounds, we demonstrate that aversive taste alone does not cause commensurate metabolic tissue loss compared to antibiotic treatment. Next, we find that mice treated with intraperitoneal antibiotics have similar loss of metabolic tissue weights as mice given oral antibiotics, despite being administered antibiotics independently of their gastrointestinal tract and thus bypassing any effect of poor taste. Finally, using germ-free mice, we show that antibiotic administration does not cause similar metabolic tissue loss in the absence of a detectable microbiome, again demonstrating that the microbiome-modulating effects of antibiotic administration, rather than food and water aversion, are driving these changes. Taken together, these findings identify food and water aversion as an important potential confounder to antibiotic use in microbiome manipulation, but also demonstrate it is not the primary mediator of metabolic tissue loss in antibiotic-treated mice. It also identifies two regimens, cefoperazone and enrofloxacin/ampicillin, which have less of an aversive effect than the commonly-used four drug regimen, although even these do not eliminate the effect. Additionally, some studies have suggested that oral antibiotic gavage may have even less of an aversive effect [9,27,28], although quantitative measures of food and water consumption and tissue-specific evaluation of these treatments are limited, requiring additional study.

We show that mice treated with several oral antibiotic regimens exhibit food and water aversion and lose metabolic tissue weight. The prior literature on this phenomenon is conflicting, which is likely due to the wide heterogeneity in the models used. It is well-known that germ-free mice gain less weight than conventional mice despite eating more [29]. Likewise, germ-free mice exhibit reduced skeletal muscle weight, which is rescued by microbiota transplantation [2]. Conversely, however, certain antibiotic treatments can be used to facilitate weight gain in domestic animals [30–32]. The reasons for this effect are unclear, but timing, dose, and spectrum of coverage of the antibiotics appear to play key roles. Domestic animals are often treated with subtherapeutic doses starting soon after birth, which facilitates weight gain across a variety of species; this effect becomes less marked with later exposures [30,33]. Additionally, human antibiotic exposure in infancy is also associated with increased risk of being overweight later in childhood [34–36]. Antibiotic dosing after this early period, or therapeutic antibiotic dosing, may result in the effects that we observed. While we did not perform paired measurement of food/water aversion and metabolic tissue weights in the same animals, we strove to minimize experimental variation across our sequential studies by using genetically-identical mice from the same vendor, identical antibiotic treatments, water, and food.

In our study, the mechanism by which antibiotics cause tissue weight loss remains unclear. Gut microbiota have been shown to be important for growth and function of skeletal muscle, and secrete metabolites that may be used by the host [2,37]. Other microbiota-produced metabolites are also important in mediating fat mass and metabolism [38]. The tested antibiotic regimens may preferentially kill off certain weight gain-promoting bacteria, promote the growth of weight loss-promoting bacteria, or a combination of the two. It is also possible that the antibiotic regimens may mediate some of these dietary and tissue weight changes through non-antimicrobial chemical activity (e.g. direct action on a receptor or cell type), although this seems less likely given the variety of chemical classes and means of administration used in these experiments. While we did not directly measure food and water consumption in antibiotic-exposed germ-free mice, the lack of difference in their overall and tissue-specific weight (Fig 7) argues against a strong microbiome-independent effect on metabolism (especially as compared to the tissue weight loss observed in mice with conventional microbiota, Fig 2). Further studies are needed to identify which key bacterial taxa, as well as the bacterial metabolites, that may mediate host changes. In addition, transcriptional analysis of the studied organs and histology may provide additional insights into the mechanisms underlying these changes.

Regardless of the underlying mechanism, this study has important implications for the use of antibiotics in experimental modulation of the microbiome. Unsurprisingly, we demonstrate that different antibiotics create distinct changes in microbial communities. Thus, antibiotic-mediated changes are not uniform, but depend on the specific regimen used. Given the wide array of regimens used in the literature [8], this can be a profound source of unintended experimental variation. Likewise, antibiotic treatment effects on food and water consumption were variable among the tested regimens. This is a potential confounder that should be considered in microbiome manipulation studies, especially since the four-drug regimen, which is among the most common models used [8], caused the greatest decrease in food and water consumption among the regimens we tested. In addition, these effects were reduced but not eliminated with milder antibiotic regimens where, unlike with the four-drug regimen, the mice still gained weight. Furthermore, while some previous work has used whole-mouse NMR spectroscopy to determine body composition [27,28], our data suggest that even when lean and fat mass is similar by this method, that this can mask considerable variability in the distribution of lean and fat mass. Taken together, our results suggest that multiple antibiotic interventions, along with evaluation of food and water consumption, should be considered to ensure robustness of findings from studies that experimentally modulate the microbiome. They further suggest that NMR spectroscopy alone is inadequate to determine mouse metabolic tissue distribution, and should be accompanied by necropsy.

Despite using high doses of bitterants known previously to be aversive [18–21], mice given bitterants in drinking water exhibited reduced water consumption, but did not eat less food. Conversely, mice given antibiotics both ate and drank significantly less, although there were no antibiotics in the mouse chow. Interestingly, the antibiotic-treated mice were more aversive than even those treated with very high levels of bitter-tasting compounds, and mice treated with intraperitoneal antibiotics were also food- and water-aversive, despite not taking in any antibiotic by mouth. This suggests that antibiotics have additional effects beyond just bad taste, and could suggest that the metabolic effects of the microbiome may be related not only to tissue metabolism, but also brain regulation of appetite and satiety. It is also interesting that bitterants alone can change microbiome composition; whether these changes are caused by reduced water consumption alone or direct factors remains unclear. Further studies are needed to clarify these effects.

## Conclusions

The present study identifies antibiotics as a mediator of tissue weight loss in mice, and identifies microbiome changes, rather than behavioral changes, as the key driver of tissue weight loss. These findings both inform experimental design in future microbiome studies and identify new avenues for research into the role of the microbiome in organismal metabolism.

## Supporting information

S1 Fig Commonly administered enteral antibiotic regimens have profound yet variable effects on host metabolism when normalized to total body weight. Mice were given an oral regimen of antibiotics: (1) 0.5 g/L cefoperazone; (2) 0.27 g/L enrofloxacin and 1 g/L ampicillin; (3) 1 g/L neomycin, 1 g/L ampicillin, 1 g/L metronidazole, and 0.5 g/L vancomycin (the “four-drug regimen”); in distilled drinking water. Control mice were given distilled drinking water alone. Mice were given *ad libitum* access to food and water. Mice were necropsied on day 8 of treatment and normalized tissue weights (tissue weight/total body weight) were calculated for liver (A), retroperitoneal fat pads (B), perigonadal fat pads (C), bilateral gastrocnemius-soleus complex muscles (D), bilateral tibialis anterior muscles (E), and bilateral kidneys (F) and compared to their respective controls. n = 10, * *P* < 0.05; N.S. = not significant, *P* > 0.05. Error bars denote SEM.
(DOCX)

S2 Fig Effects of oral antibiotic regimens on bacterial family composition in the mouse cecum. Mice were administered enteral antibiotics via distilled drinking water (cefoperazone, enrofloxacin/ampicillin, or the four-drug regimen); control mice were administered distilled drinking water alone. Mice were provided *ad libitum* access to food and water and harvested on day 8. n = 30 for control, n = 10 for antibiotic treatments. Error bars denote SEM.
(DOCX)

## Abbreviations

ddPCR: droplet digital PCR
DNA: deoxyribonucleic acid
GSC: gastrocnemius-soleus complex
OTU: operational taxonomic unit
PCR: polymerase chain reaction
PERMANOVA: permutational analysis of variance
rpm: revolutions per minute
rRNA: ribosomal ribonucleic acid
SEM: standard error of the mean
TA: tibialis anterior
