# Supplementary material for: Antibiotics cause metabolic changes in mice primarily through microbiome modulation rather than behavioral changes
Source: PLoS One. 2022 Mar 17;17(3):e0265023. doi: 10.1371/journal.pone.0265023 (PMC8929607; doi:10.1371/journal.pone.0265023)
Supplement: S1 Fig — Mice were given an oral regimen of antibiotics: (1) 0.5 g/L cefoperazone; (2) 0.27 g/L enrofloxacin and 1 g/L ampicillin; (3) 1 g/L neomycin, 1 g/L ampicillin, 1 g/L metronidazole, and 0.5 g/L vancomycin (the “four-drug regimen”); in distilled drinking water. Control mice were given distilled drinking water alone. Mice were given ad libitum access to food and water. Mice were necropsied on day 8 of treatment and normalized tissue weights (tissue weight/total body weight) were calculated for liver (A), retroperitoneal fat pads (B), perigonadal fat pads (C), bilateral gastrocnemius-soleus complex muscles (D), bilateral tibialis anterior muscles (E), and bilateral kidneys (F) and compared to their respective controls. n = 10, * P < 0.05; N.S. = not significant, P > 0.05. Error bars denote SEM. (DOCX) [file pone.0265023.s001.docx]

**
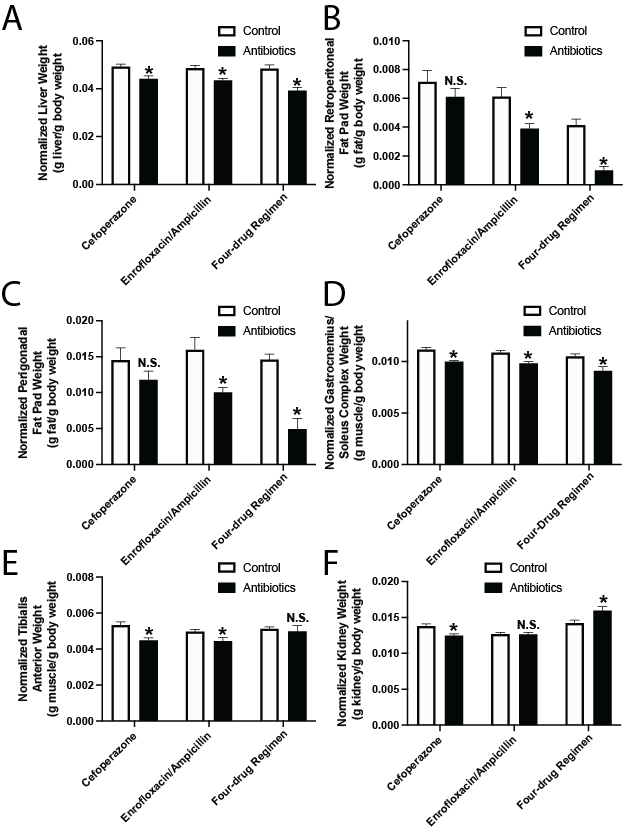
Supplemental Figure 1:** Commonly administered enteral antibiotic regimens have profound yet variable effects on host metabolism when normalized to total body weight. Mice were given an oral regimen of antibiotics: (1) 0.5 g/L cefoperazone; (2) 0.27 g/L enrofloxacin and 1 g/L ampicillin; (3) 1 g/L neomycin, 1 g/L ampicillin, 1 g/L metronidazole, and 0.5 g/L vancomycin (the “four-drug regimen”); in distilled drinking water. Control mice were given distilled drinking water alone. Mice were given *ad libitum* access to food and water. Mice were necropsied on day 8 of treatment and normalized tissue weights (tissue weight/total body weight) were calculated for liver (A), retroperitoneal fat pads (B), perigonadal fat pads (C), bilateral gastrocnemius-soleus complex muscles (D), bilateral tibialis anterior muscles (E), and bilateral kidneys (F) and compared to their respective controls. n=10, * *P* < 0.05; N.S.=not significant, *P* > 0.05. . Error bars denote SEM.
