# Supplementary material for: Antibiotics cause metabolic changes in mice primarily through microbiome modulation rather than behavioral changes
Source: PLoS One. 2022 Mar 17;17(3):e0265023. doi: 10.1371/journal.pone.0265023 (PMC8929607; doi:10.1371/journal.pone.0265023)
Supplement: S2 Fig — Mice were administered enteral antibiotics via distilled drinking water (cefoperazone, enrofloxacin/ampicillin, or the four-drug regimen); control mice were administered distilled drinking water alone. Mice were provided ad libitum access to food and water and harvested on day 8. n = 30 for control, n = 10 for antibiotic treatments. Error bars denote SEM. (DOCX) [file pone.0265023.s002.docx]

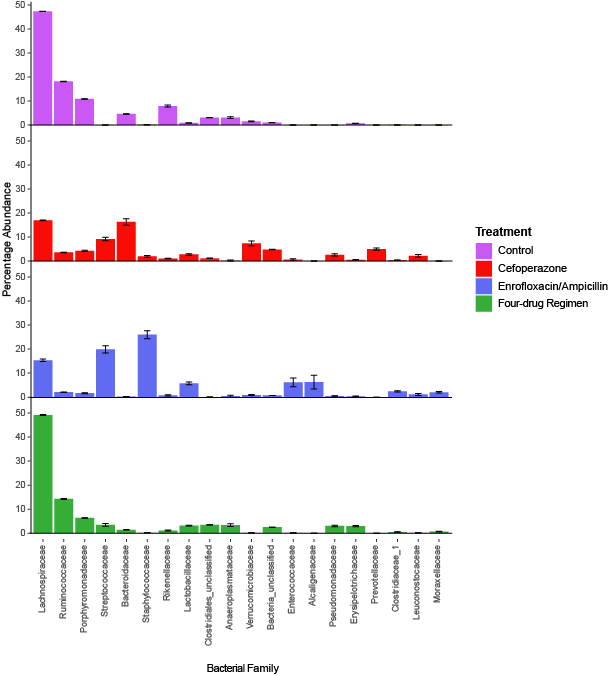


**Supplemental Figure 2:** Effects of oral antibiotic regimens on bacterial family composition in the mouse cecum. Mice were administered enteral antibiotics via distilled drinking water (cefoperazone, enrofloxacin/ampicillin, or the four-drug regimen); control mice were administered distilled drinking water alone. Mice were provided *ad libitum* access to food and water and harvested on day 8. n=30 for control, n=10 for antibiotic treatments. Error bars denote SEM.
